# Morphofunctional State of the Liver Under Conditions of Three-Month Dark Deprivation: The Influence of Circadian Disruptions and Melatonin

**DOI:** 10.3390/ijms27062663

**Published:** 2026-03-14

**Authors:** David A. Areshidze, Maria A. Kozlova, Anna I. Anurkina, Valeriy P. Chernikov

**Affiliations:** Avtsyn Research Institute of Human Morphology of Petrovsky National Research Centre of Surgery, 117418 Moscow, Russia

**Keywords:** dark deprivation, constant lighting, circadian rhythms, desynchronosis, melatonin, liver, hepatocytes, aging, steatosis

## Abstract

Disruption of circadian rhythms caused by constant artificial lighting (“light pollution”) is a significant risk factor for the development of metabolic and age-associated pathologies. The liver, as a central metabolic organ with pronounced circadian regulation of its functions, is particularly vulnerable to desynchronosis. The aim of this study was to evaluate the effect of three-month dark deprivation (constant lighting) and the corrective action of exogenous melatonin on the morphofunctional state of the liver in young mature rats. The experiment used 3-month-old male Wistar rats, divided into groups: control (standard light:dark cycle 10:14 h), dark deprivation group (DD, constant lighting 24 h/day), and DD + Melatonin group (DD + Mel, dark deprivation with melatonin administered in drinking water at a dose of 12 mg/L). After 3 months (animal age 6 months), a comprehensive analysis was performed. It was shown that dark deprivation causes a profound (more than five-fold) suppression of plasma melatonin levels, which is accompanied by the formation of a pro-senescent and metabolically dysfunctional phenotype of the liver. This was manifested by the development of steatosis, an 18% increase in hepatocyte area, a 30% decrease in the proportion of binucleated hepatocytes, activation of cellular senescence markers (p16, p21) and stress markers (p53), and suppression of the expression of circadian transcription factors BMAL1 and CLOCK. At the ultrastructural level, lipofuscin accumulation, damage to mitochondria and the Golgi apparatus were noted. Biochemically, hyperglycemia, increased AST activity, hypoproteinemia, hypoalbuminemia, hypercholesterolemia, and hypertriglyceridemia were revealed. Administration of exogenous melatonin completely prevented the development of these disorders, normalizing hormone levels, morphology, ultrastructure, biochemical parameters, and the expression of key molecular markers. Thus, three-month dark deprivation induces complex pro-senescent remodeling and metabolic dysfunction of the liver, mediated by melatonin deficiency, while exogenous melatonin demonstrates a pronounced hepatoprotective and chronoprotective effect.

## 1. Introduction

The modern urban environment is characterized by an unprecedented level of light pollution. According to the International Dark-Sky Association, over 80% of the world’s population lives under skies where natural day–night cycles are substantially distorted by artificial lighting [1,2]. Night shift work, the use of light-emitting screens (smartphones, tablets, computers) during evening and nighttime hours, and the 24/7 illumination of megacities have rendered chronic desynchronosis an integral attribute of the modern lifestyle. As early as 2007, the World Health Organization classified night shift work as a probable carcinogenic factor (Group 2A) [3,4]. However, over the past years, compelling evidence has accumulated that the spectrum of pathological consequences of desynchronosis is considerably broader, encompassing obesity, type 2 diabetes mellitus, cardiovascular diseases, neurodegenerative disorders, and accelerated organismal aging [2,5].

While the core principles of circadian biology—endogenous, entrainable oscillations with a period of approximately 24 h—are conserved across kingdoms, the underlying molecular mechanisms exhibit fascinating diversity. In plants, the circadian system is decentralized, with most cells containing an autonomous oscillator that is primarily light-entrained, coordinating essential processes like photosynthesis, flowering, and stomatal opening [6,7]. In contrast, animals have evolved a hierarchically organized system, with a master pacemaker in the brain (the suprachiasmatic nuclei) that synchronizes peripheral oscillators in organs like the liver, heart, and kidney through neural and hormonal signals, most notably melatonin. Despite these architectural differences, both kingdoms share conserved molecular feedback loops involving transcription-translation, demonstrating the fundamental evolutionary importance of anticipating daily environmental changes [8,9].

The mammalian circadian system represents a hierarchically organized structure. The central oscillator is localized in the suprachiasmatic nuclei (SCN) of the hypothalamus and is synchronized by external light signals via the retinohypothalamic tract [10,11]. Peripheral oscillators are present in virtually all cells of the body, including hepatocytes, cardiomyocytes, adipocytes, and pancreatic cells. At the molecular level, clock function is ensured by autonomous transcription–translation feedback loops. The heterodimer of transcription factors BMAL1 (ARNTL) and CLOCK binds to E-box elements in the promoters of Period (Per1, Per2, Per3) and Cryptochrome (Cry1, Cry2) genes, activating their expression [12,13,14]. PER and CRY proteins, accumulating in the cytoplasm, form complexes, translocate to the nucleus, and inhibit BMAL1/CLOCK activity, thereby suppressing their own transcription. The cycle is completed by PER/CRY degradation and resumption of transcription. Additional feedback loops involving nuclear receptors REV-ERBα/β and RORα modulate the amplitude and period of oscillations [15,16].

The key link converting the light signal into an endocrine output is the pineal gland. In darkness, under the influence of norepinephrine released from postganglionic sympathetic fibers, the enzyme aralkylamine N-acetyltransferase (AANAT) is activated in pinealocytes, converting serotonin into N-acetylserotonin, which is further metabolized into melatonin (N-acetyl-5-methoxytryptamine) by hydroxyindole-O-methyltransferase (HIOMT) [17]. Light inhibits AANAT activity, leading to a rapid decrease in hormone levels. Melatonin enters the bloodstream and cerebrospinal fluid, delivering photoperiodic information to all body cells. Beyond its chronobiotic function, melatonin is a potent endogenous antioxidant, immunomodulator, apoptosis inhibitor, and geroprotector. Its receptors (MT1 and MT2) belong to the G-protein-coupled receptor family and are abundantly expressed in the liver, kidneys, heart, and blood vessels [18].

The liver occupies a special position among peripheral oscillators. Up to 80–90% of genes encoding proteins of intermediary metabolism in hepatocytes are subject to circadian regulation [19,20,21]. Moreover, the hepatic parenchyma exhibits pronounced daily fluctuations not only at the molecular but also at the tissue level: organ volume, xenobiotic detoxification rate, synthesis of albumin, cholesterol, and bile acids, and the activity of glycolysis and gluconeogenesis enzymes vary with time of day [22,23]. BMAL1 regulates the expression of phosphoenolpyruvate carboxykinase (PEPCK) and glucose-6-phosphatase (G6Pase), controlling gluconeogenesis. Mice with liver-specific Bmal1 knockout lose the ability to maintain normoglycemia during the rest phase. The clock controls the activity of sterol regulatory element-binding protein 1 (SREBP1), acetyl-CoA carboxylase (ACC), and fatty acid synthase (FASN). Deletion of Cry genes results in hypertriglyceridemia and steatosis. The expression of many cytochrome P450 isoforms (CYP2E1, CYP3A4, CYP7A1) is under circadian control, which explains the chronopharmacological effects of drugs [24,25,26,27,28,29,30,31,32,33,34,35,36].

Thus, the liver represents an ideal model for studying the impact of desynchronosis on metabolic health. The most adequate experimental model for investigating the consequences of chronic light pollution is housing laboratory animals under constant light conditions (24 h light, 0 h darkness), also termed “dark deprivation”. This regimen leads to elimination of daily rhythmicity in behavior and physiological functions; profound and sustained suppression of endogenous melatonin synthesis (by 80–90% of baseline); desynchronization of molecular clocks in the SCN and peripheral tissues; and oxidative stress due to the loss of antioxidant protection provided by melatonin [37,38,39,40,41,42].

Importantly, unlike models involving complete pinealectomy, dark deprivation is functional, reversible, and ethically more acceptable as it does not require surgical intervention.

The relationship between desynchronosis and accelerated aging has been intensively investigated over the past decade. It has been shown that in elderly individuals, the amplitude of circadian rhythms is reduced, and nighttime melatonin levels are significantly lower than in young subjects. In experimental models, constant lighting accelerates the appearance of age-associated phenotypes: sarcopenia, osteoporosis, thymic involution, and cognitive deficits. However, data on the comprehensive impact of long-term dark deprivation specifically on the liver, with emphasis on markers of cellular senescence and the circadian status of hepatocytes, remain insufficient in the literature. Most studies are confined either to biochemical analysis or to isolated molecular pathways, failing to provide an integrative picture. Given that the central pathogenic link in dark deprivation is endogenous melatonin deficiency, exogenous hormone administration is regarded as a pathogenetically justified corrective strategy. Numerous studies have confirmed that melatonin at doses of 10–20 mg/L of drinking water can: restore circadian rhythm amplitude; exert antioxidant and anti-inflammatory effects; improve mitochondrial function; suppress activation of senescence pathways (p53/p21, p16INK4a/Rb) [43,44,45,46,47,48,49].

However, the question of whether a long-term (three-month) course of melatonin can completely prevent the formation of a pro-senescent hepatic phenotype in young animals under conditions of permanent light stress remains open.

We hypothesized that chronic dark deprivation induces a complex of structural and metabolic changes in the liver that meet the criteria of accelerated aging (hypertrophy, polyploidization, steatosis, mitochondrial dysfunction, elevated p16/p21, reduced regenerative potential), with melatonin deficiency serving as the key trigger, and that exogenous melatonin, by replenishing this deficiency, is capable of blocking the entire cascade of pathological reactions.

Despite active research into the relationship between desynchronosis and aging, data on the comprehensive impact of long-term dark deprivation specifically on the liver, with emphasis on markers of cellular senescence and the circadian status of hepatocytes, remain insufficient in the literature. Most studies are confined either to biochemical analysis or to isolated molecular pathways, failing to provide an integrative picture that spans from systemic hormone levels to ultrastructural organization. Furthermore, the question of whether a long-term (three-month) course of melatonin can completely prevent the formation of a pro-senescent hepatic phenotype in young animals under conditions of permanent light stress remains open. The novelty of the present study lies in its multi-level, integrative approach—combining biochemical, histological, ultrastructural, and molecular analyses—to demonstrate for the first time that chronic dark deprivation induces a complex of structural and metabolic changes in the liver that meet the criteria of accelerated aging, with melatonin deficiency serving as the key trigger. We hypothesized that exogenous melatonin, by replenishing this deficiency, is capable of blocking the entire cascade of pathological reactions. Therefore, the aim of the present study was a comprehensive evaluation of the effects of three-month dark deprivation and the corrective action of exogenous melatonin on the morphofunctional, ultrastructural, and molecular state of the liver in young adult rats.

## 2. Results

### 2.1. Melatonin Level and Biochemical Parameters

Three-month dark deprivation induced a profound suppression of plasma melatonin levels: in the DD group, its concentration (15.18 ± 3.27 pg/mL) was more than 5 times lower than in the control group (77.29 ± 8.59 pg/mL, *p* ≤ 0.0005). In the DD + Mel group, the hormone level (65.58 ± 11.90 pg/mL) did not differ from the control (Table 1). Animals in the DD group exhibited a complex of biochemical disorders: a significant decrease in glucose level (hypoglycemia), increased AST activity (a marker of cytolysis), markedly reduced concentrations of total protein and albumin (hypoproteinemia and hypoalbuminemia), as well as elevated levels of cholesterol and triglycerides. LDH and ALP activities were also increased. Total and direct bilirubin levels showed no significant differences from the control. In the DD + Mel group, all of the aforementioned biochemical parameters were normalized and did not statistically differ from the control values (Table 1).

### 2.2. Liver Morphology and Morphometry

**Figure 1:**
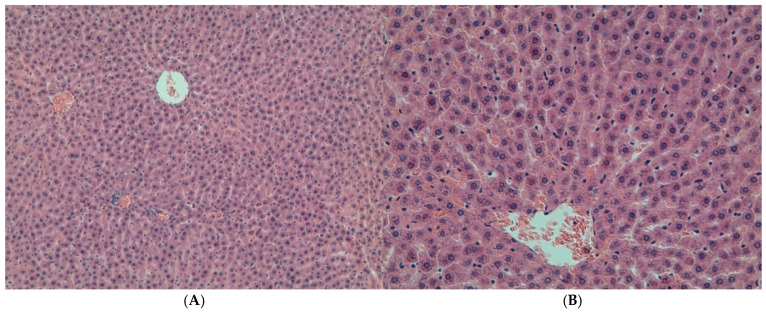
Liver of rats from the control group. Hematoxylin and eosin staining, (**A**)—×200, (**B**)—×400.

**Figure 2:**
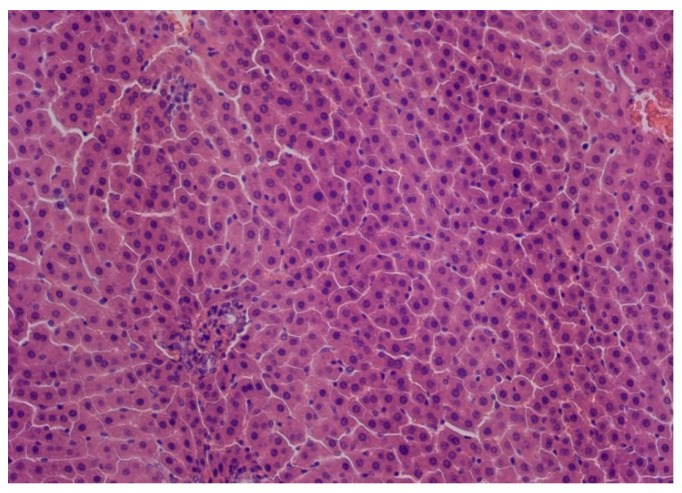
Liver of rats from the DD + MEL group. Hematoxylin and eosin staining, ×200.

**Figure 3:**
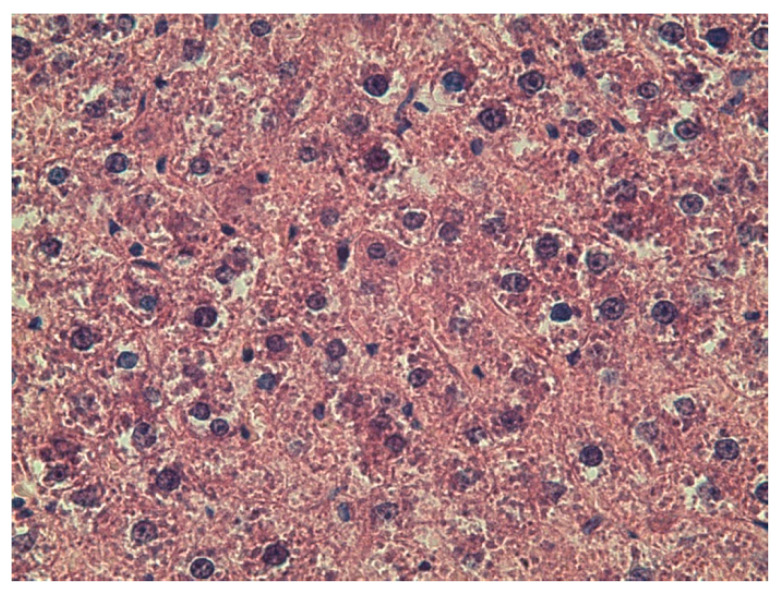
Liver of rats from the DD group. Hematoxylin and eosin staining, ×400.

Microscopic examination of the liver in the control group and the DD + Mel group revealed normal histological structure: the lobular architecture was preserved, and polygonal hepatocytes formed distinct hepatic cords (Figure 1A,B and Figure 2). In the DD group, while the general structure of the organ was preserved, changes were observed in the form of hydropic and microvesicular fatty degeneration of hepatocytes, as well as single necrotic cells (Figure 3).

The proportion of binuclear hepatocytes was significantly lower in the DD group compared to the control. In contrast, the DD + Mel group exhibited a significantly higher proportion of binuclear cells compared to both the control and the DD group. The mean ploidy of hepatocytes tended to increase in both the DD and DD + Mel groups relative to the control; however, the differences were not statistically significant due to high data variability.

The proportion of connective tissue was significantly increased in the DD group compared to the control, indicating the development of fibrosis. In the DD + Mel group, this parameter returned to control values.

The proportion of hepatocytes with fatty degeneration was significantly higher in the DD group than in the control. Although this parameter was significantly reduced in the DD + Mel group compared to the DD group, it remained slightly but significantly elevated relative to the control.

The DD group exhibited a pronounced degree of steatosis, which was significantly higher than in the control. However, in the DD + Mel group, steatosis was significantly less pronounced compared to the DD group and did not differ from the control (Table 2).

### 2.3. Molecular Marker Expression

Immunohistochemical analysis revealed significant changes in the DD group (Table 3). A sharp increase in the expression of cellular senescence markers p16 (24.2-fold) and p21 (6.8-fold), as well as the stress-associated protein p53 (1.8-fold), was observed compared to the control. Concurrently, a profound suppression of key circadian oscillator activators—BMAL1 and CLOCK—was recorded, while PER2 expression was significantly elevated. The Ki-67 proliferation index was also significantly increased in the DD group, indicating compensatory hyperplasia in response to damage. In the DD + Mel group, the expression of all studied markers (p16, p21, p53, BMAL1, CLOCK, PER2, Ki-67) did not differ from control values, indicating a complete normalization of the molecular profile of hepatocytes under the influence of melatonin.

### 2.4. Ultrastructural Organization of Hepatocytes

**Figure 4:**
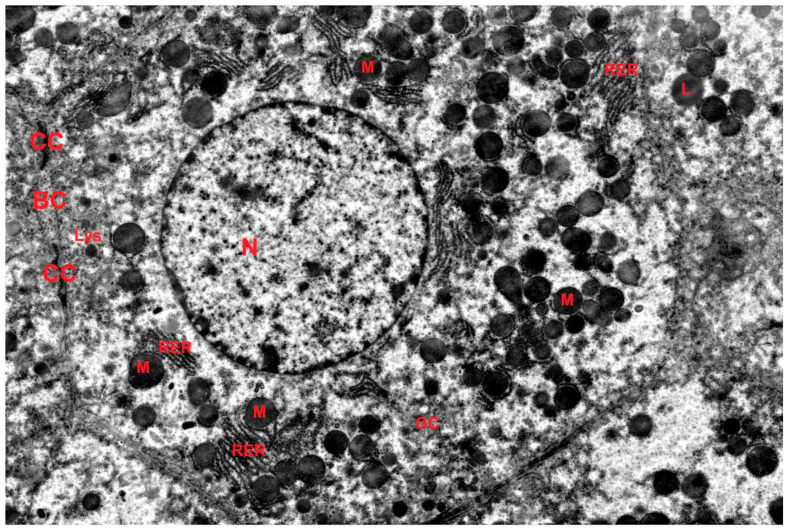
Ultrastructure of hepatocytes of animals of the control group, RER—rough endoplasmic reticulum, BC—bile canaliculi, GC—Golgi complex, CC—cell–cell contact, L—lipid droplet, Lys—lysosome, M—mitochondrion, N—nucleus. TEM, ×5500.

**Figure 5:**
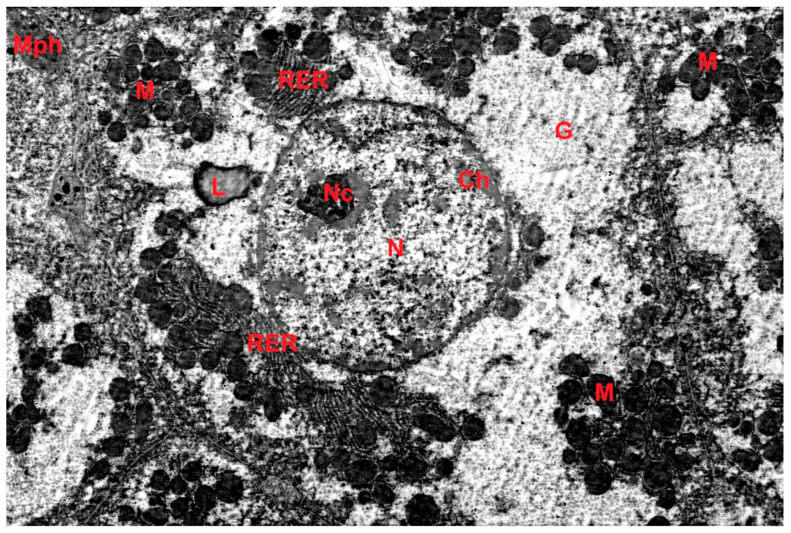
Ultrastructure of hepatocytes of animals of the DD group. G—glycogen, RER—granular endoplasmic reticulum, L—lipid droplet, M—mitochondria, Mph—macrophage, Ch—chromatin, N—nucleus, Nc—nucleolus. TEM, ×5000.

**Figure 6:**
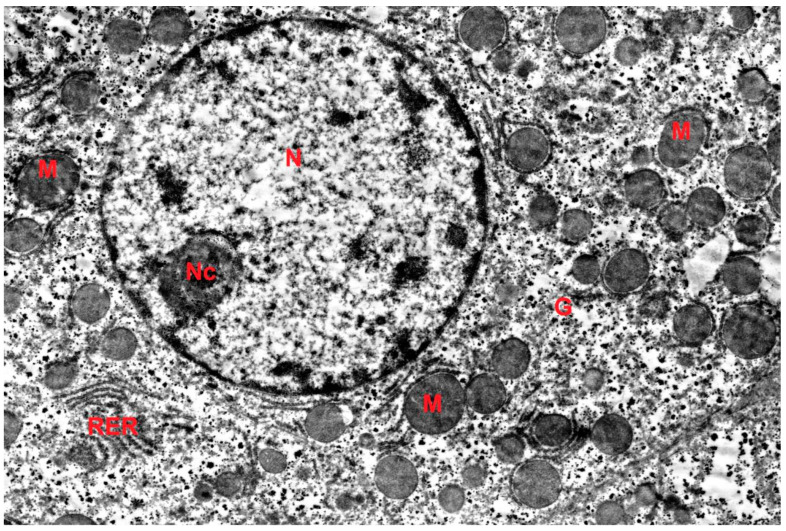
Ultrastructure of hepatocytes of animals of the DD + MEL group. G—glycogen, RER—rough endoplasmic reticulum, M—mitochondrion, N—nucleus, Nc—nucleolus. TEM, ×8000.

The ultrastructure of hepatocytes in the control group corresponded to the norm (Figure 4). Electron microscopic examination in the DD group revealed signs of accelerated aging: accumulation of lipofuscin pigment in hepatocytes, polymorphism and swelling of mitochondria with cristae destruction, dilation of granular endoplasmic reticulum (GER) cisternae, and reduction of the Golgi complex (Figure 5). Morphometry showed a decrease in the numerical density of mitochondria, a reduction in the number of cristae, and a decrease in the profile area of the Golgi complex in the DD group compared to the control. In the DD + Mel group, the ultrastructure of hepatocytes was comparable to the control: rounded nuclei, mitochondria with dense matrix, well-developed GER, and numerous dictyosomes of the Golgi complex were observed (Figure 6).

Morphometric analysis of mitochondria (Table 4) showed that in the DD group, compared to the control, there was a trend toward a decrease in the numerical density of mitochondria and a significant reduction in the number of cristae in mitochondria. The cross-sectional area and perimeter of the organelles did not change significantly; however, the circularity index was slightly higher than in the control, which may indicate initial stages of swelling. In the DD + Mel group, all mitochondrial parameters did not differ from control values and were significantly higher than in the DD group for a number of indicators (numerical density, number of cristae).

Morphometric analysis of the Golgi complex (Table 5) revealed a trend toward a decrease in all studied parameters in the DD group compared to the control, although the differences did not reach statistical significance. The most pronounced changes were a reduction in the number of cisternae in dictyosomes (by 12.3%) and a decrease in the profile area of the Golgi complex (by 15.9%). In the DD + Mel group, all parameters corresponded to control levels (Table 5).

## 3. Discussion

The present study provides a comprehensive characterization of structural and functional changes in the rat liver under conditions of chronic light deprivation (constant illumination for three months) and demonstrates a pronounced protective effect of exogenous melatonin. The obtained data indicate that desynchronosis, induced by suppression of endogenous melatonin secretion, initiates a cascade of pathological processes at the systemic, tissue, cellular, and molecular levels, the totality of which meets the criteria for accelerated liver aging.

Three-month housing under constant lighting conditions led to a significant decrease in plasma melatonin levels, confirming the adequacy of the selected model for studying the consequences of light pollution. It is known that light inhibits the activity of AANAT (aralkylamine N-acetyltransferase) in pinealocytes, which is a key mechanism for suppressing the nocturnal melatonin peak [50]. The review by Srinivasa et al. [51] emphasizes that the liver functions as a peripheral clock closely connected to the suprachiasmatic nucleus (SCN), and disturbances in circadian rhythms are directly associated with the development of metabolic dysfunction-associated steatotic liver disease (MASLD) and hepatocellular carcinoma (HCC). Importantly, melatonin interacts with nuclear receptors ROR-α (retinoic acid receptor-related orphan receptor-alpha), which are critical for maintaining circadian rhythms and possess anti-inflammatory and potentially anti-tumor properties [52]. Chronic melatonin suppression in our model thus creates conditions for multiple disturbances in hepatocellular homeostasis through dysregulation of both receptor-mediated (MT1/MT2, ROR-α) and non-receptor mechanisms.

Modern humans face a dual effect of the artificial light environment: on one hand, excessive nighttime illumination (ALAN) suppresses melatonin synthesis, and on the other hand, lack of daytime sunlight (especially its blue-violet spectrum) disrupts circadian system synchronization and leads to vitamin D deficiency. This combination creates a “perfect storm” for the development of so-called “diseases of civilization,” including metabolic syndrome and liver pathology. Our experimental data fully confirm this concept at the tissue and molecular levels [53].

The identified biochemical shifts in animals of the DD group (hypoglycemia, hypoproteinemia, hypoalbuminemia, hypercholesterolemia, hypertriglyceridemia, increased AST, ALT, LDH, and ALP) indicate profound metabolic disorders and hepatocyte damage. These data are consistent with current understanding of the role of circadian dysregulation in the pathogenesis of MASLD [54,55,56].

Increased activity of AST, ALT, and LDH in the DD group is a marker of cytolysis and indicates damage to hepatocyte membranes, which at the ultrastructural level is confirmed by mitochondrial swelling and cristae destruction. Complete normalization of all biochemical parameters in the DD + Mel group underscores the key role of melatonin in maintaining hepatic metabolic homeostasis.

The increase in hepatocyte area by 42.5% with unchanged nuclear area and the decrease in N/C ratio by 30.4% in the DD group indicate cellular hypertrophy. Hepatocyte hypertrophy is often considered an adaptive response to metabolic stress; however, it can also be a precursor to pathological remodeling and fibrosis [57,58,59].

A twofold increase in the proportion of connective tissue confirms the development of fibrosis. The work of Kim and Cheon [60] sheds light on the molecular mechanisms of the antifibrotic action of melatonin. The authors showed that melatonin, through activation of the MT2 receptor, increases the expression of BMAL1 and antioxidant enzymes, suppressing TGF-β1-induced activation of hepatic stellate cells (HSCs). The use of an MT2 antagonist or siRNA against MT2 completely abolished these effects, proving that the MT2-BMAL1 signaling pathway is critical for the antifibrotic action of melatonin. In our model, fibrosis induced by light deprivation was completely prevented by melatonin, which is consistent with the described mechanism. Pramong et al. [61] also demonstrated that in aged rats, the expression of collagen type I in the liver increases, and melatonin treatment reduces this parameter.

Pronounced steatosis in the DD group and an increase in the proportion of hepatocytes with fatty degeneration confirm that light pollution is a risk factor for the development of MASLD. Melatonin, by restoring circadian regulation of lipid metabolism and possessing antioxidant properties, significantly reduced steatosis in the DD + Mel group (0.90 points). The meta-analysis by Singh [62] confirms these data, demonstrating clinically significant improvement in the lipid profile of patients with MASLD during melatonin administration. Nevertheless, the proportion of hepatocytes with fatty degeneration in our work remained slightly but significantly elevated relative to the control, which may indicate the need for longer therapy for complete elimination of lipid inclusions or the existence of a pool of cells with irreversible changes [63].

The decrease in the proportion of binuclear hepatocytes in the DD group may reflect disruption of the cytokinesis process or depletion of the pool of cells capable of forming binuclear forms, which are considered a reserve for polyploidization and regeneration [64]. Work using BTBR mice [65] clearly demonstrated that three populations of cells are present in the liver: mononuclear diploid, binuclear, and mononuclear tetraploid, and their ratio is an important indicator of the functional state of the organ. Interestingly, in the DD + Mel group, the proportion of binuclear cells was significantly higher not only compared to DD but also to the control. This may indicate activation of regenerative processes and an increase in the functional reserve of the liver under the influence of melatonin [66].

The trend toward an increase in mean ploidy in both experimental groups (to 3.22 and 3.20 n) is consistent with the concept of stress-induced polyploidization as an adaptive mechanism allowing hepatocytes to increase functional capacity without division [67]. The paradoxical increase in the Ki-67 proliferation index in the DD group against the background of pronounced activation of cellular senescence markers (p16, p21) may be explained by compensatory hyperplasia in response to chronic damage and cell death. However, such proliferation is likely “unbalanced” and does not lead to effective restoration of the population of functionally competent hepatocytes. Normalization of Ki-67 in the DD + Mel group indicates stabilization of cell renewal and elimination of chronic damage [68,69].

The most significant result of this work is the demonstration that chronic light deprivation induces a cellular senescence phenotype in the liver, as evidenced by a sharp increase in the expression of p16 and p21, as well as p53. p16 and p21 are universally recognized biomarkers of aging, and their expression is minimal in young tissues [70,71]. González-Gallego et al. [72], in a series of works, demonstrated that melatonin is capable of reducing apoptotic changes in the liver of aging rats through inhibition of the mitochondrial apoptosis pathway and preventing oxidative stress. Activation of these pathways in our study indicates the initiation of cellular senescence programs in a significant portion of hepatocytes.

Simultaneous profound suppression of BMAL1 and CLOCK in the DD group demonstrates the disintegration of the molecular circadian oscillator. A study in mice [73] showed that Clock mutation leads to arrhythmic expression of Per1 and Per2 in the liver and skeletal muscle, despite preserved rhythms in the SCN, emphasizing the tissue-specific requirements for clock components and the absolute dependence of peripheral oscillators on functional CLOCK. In our model, suppression of endogenous melatonin led to a similar effect—desynchronization of hepatic clocks with preserved (presumably) SCN function. The increase in PER2 in the DD group may be a consequence of disruption of feedback mechanisms in the circadian cycle.

The complete normalization of all molecular markers (p16, p21, p53, BMAL1, CLOCK, PER2, Ki-67) in the DD + Mel group indicates that exogenous melatonin not only compensates for the deficiency of the endogenous hormone but also restores the regulatory circuits controlling aging and circadian rhythmicity, likely through receptor-mediated mechanisms, including the MT2-BMAL1 axis [74,75,76] and modulation of ROR-α activity [77]. Indeed, it has been shown that melatonin, through interaction with membrane receptors, modulates the expression of clock genes, including BMAL1 and CLOCK, in liver cells, and that nuclear ROR-α receptors, also under the control of melatonin, are critically important for maintaining circadian rhythms and possess anti-inflammatory properties.

Moreover, fundamental studies demonstrate a direct link between clock genes (BMAL1, CLOCK, PER2) and the regulation of aging markers p16, p21, and p53, which explains the complete normalization of these parameters in our model upon restoration of circadian rhythmicity by exogenous melatonin [78,79,80,81].

Electron microscopy data provided direct visualization of the damage underlying the observed functional disturbances. Accumulation of lipofuscin—the “aging pigment”—is a marker of oxidative stress and reduced autophagy efficiency [82,83,84]. The study by Manikonda et al. [85] showed that with age (in 12- and 24-month-old rats), lipid peroxidation increases in the liver, the GSH/GSSG ratio and the activity of antioxidant enzymes decrease, and the daily rhythms of these parameters are disrupted. Melatonin administration partially restored the amplitude and acrophases of these rhythms. In our study, young (6-month-old) rats subjected to light deprivation exhibited ultrastructural changes similar to age-related ones, confirming the concept of “accelerated aging” under the influence of desynchronosis.

Mitochondrial damage (polymorphism, swelling, cristae destruction, tendency to decreased numerical density) confirms the key role of mitochondrial dysfunction in pathogenesis. Yang et al. [86], in a model of atrazine-induced toxic liver damage, showed that melatonin restores the level of Rab8a—a protein critical for regulating fatty acid utilization in mitochondria. Rab8a knockdown abolished the protective effect of melatonin, indicating a new molecular mechanism of mitoprotection. The ability of melatonin to accumulate in mitochondria at high concentrations and its direct antioxidant activity explain the preservation of organelle ultrastructure in the DD + Mel group [87].

Reduction of the Golgi complex (decreased number of cisternae and profile area) in the DD group correlates with hypoalbuminemia and impaired liver secretory function. Restoration of Golgi complex morphology under the influence of melatonin underscores its role in maintaining not only the energy but also the secretory apparatus of the cell [88,89,90].

The totality of the obtained data allows us to propose the following sequence of events: chronic light exposure, melatonin suppression, disruption of receptor signaling (MT2, ROR-α), disintegration of circadian oscillators in hepatocytes (decrease in BMAL1/CLOCK), metabolic dysregulation (dyslipidemia, impaired carbohydrate and protein metabolism), oxidative stress and mitochondrial dysfunction (decrease in Rab8a, antioxidant enzymes), activation of cellular senescence pathways (p53/p21, p16), hepatocyte hypertrophy and polyploidization, steatosis and fibrosis (through HSC activation), compensatory proliferation (increase in Ki-67) against the background of depleted regenerative reserve (decrease in proportion of binuclear cells).

Exogenous melatonin, by replenishing the hormone deficiency, acts at several levels:

- Systemic: restores circadian signaling and synchronization of peripheral oscillators;
- Receptor: activates the MT2-BMAL1-antioxidant pathway [60] and modulates ROR-α activity;
- Molecular: normalizes clock gene expression, suppresses senescence pathways (p53/p21, p16), and restores expression of mitochondrial proteins (Rab8a);
- Cellular: maintains mitochondrial homeostasis and autophagy, increases the pool of binuclear hepatocytes;
- Tissue: prevents steatosis and fibrosis.

The obtained results have direct relevance to human health. Given that more than 80% of the world’s population lives under conditions of light pollution [86], and the prevalence of MASLD reaches 38% in the population, the identification of melatonin as an effective agent for the prevention and correction of desynchronosis-associated liver damage opens new therapeutic horizons. Moreover, clinical studies are currently investigating the effect of melatonin on sleep and cognitive functions in patients with liver cirrhosis and hepatic encephalopathy. This underscores the growing interest in the therapeutic potential of melatonin in severe liver diseases and expands the range of possible clinical applications beyond metabolic disorders.

Thus, our study not only confirms the concept of light pollution as a factor in accelerated liver aging but also details the molecular, cellular, and ultrastructural mechanisms of this process, and demonstrates the powerful protective potential of melatonin, paving the way for clinical studies in at-risk groups. The obtained results have direct relevance to human health, paving the way for clinical studies in night shift workers, residents of megacities, and patients with MASLD.

## 4. Materials and Methods

### 4.1. Object of Study

This study was conducted on 90 male rats of Wistar outbred stock (3 months old).

Animals were taken from the Research and Production Enterprise “Laboratory Animal Nursery ‘Pushchino’ of Branch of the FSBIS Shemyakin and Ovchinnikov Institute of Bioorganic Chemistry of the Russian Academy of Sciences” (IBCh RAS). All animals were kept under standard vivarium conditions, with ad libitum access to drinking water and briquetted food. Initially, all of the rats were kept under natural daylight. The care of the animals and the experimental procedures were conducted in accordance with the European Convention for the Protection of Vertebrate Animals used for Experimental and other Scientific Purposes (Strasbourg, 18 March 1986). The research was approved by the Bioethical Committee of the Avtsyn Research Institute of Human Morphology of the Federal state budgetary scientific institution “Petrovsky National Research Centre of Surgery”, protocol No. 27/3 dated 11 October 2021.

### 4.2. Study Design

This study was conducted on 90 male rats of Wistar outbred stock (3 months old). At the age of 3 months, the rats were divided into 3 equal groups.

The control group (C) (n = 30) was kept under a fixed light regime (light/dark ratio of 12:12 h with lights on at 8:00 and off at 20:00) for three months.

Group 2 (Dark deprivation, DD) (n = 30) received constant lighting (24 h/day) for three months.

Group 3 (Dark deprivation + Melatonin, DD + Mel) (n = 30) received dark deprivation + melatonin (Sigma, St. Louis, MO, USA) at a dose of 12 mg/L of drinking water, administered at the beginning of the subjective night 5 times per week for three months [91,92].

Total liver preparations were used to obtain imprints, which were then stained with fuchsin-sulfurous acid according to the Feulgen method.

The ploidy of hepatocytes was calculated in ploidy units relative to the optical density of the stained diploid nuclei of small lymphocytes.

All animals were provided with standard laboratory chow and water ad libitum throughout the experiment. The duration of the experiment was three months, after which the animals reached the age of 6 months.

### 4.3. Sample Collection

At the end of the experimental period, animals were euthanized by decapitation under light ether anesthesia. Blood was collected into tubes with heparin for plasma separation. Liver tissue was rapidly excised, weighed, and processed for histological, immunohistochemical, and electron microscopic examination.

Euthanasia was carried out three weeks after the start of the experiment by decapitation using a guillotine. Animals were removed from the experiment using the time series method (regular time intervals) four times a day, at 9:00, 15:00, 21:00, and 3:00, with 7–8 animals sacrificed at each time point to ensure the statistical validity of the results. After sacrifice, evisceration was performed.

Decapitation is preferred over chemical euthanasia in studies of this type for two reasons. First of all, chemical euthanasia methods modulate the measurements of biochemical parameters of blood plasma and serum in comparison with the norm (for example, CO_2_ causes acidosis, and potassium chloride prevents analysis of serum potassium ion levels); moreover, anesthetic agents can directly affect tissue viability or parameters. Chemical agents may directly damage tissues (for example, intraperitoneal alcohol and intraperitoneal pentobarbital both diminish the tinctorial qualities in histologic sections). Of all euthanasia methods, decapitation is the most consistent with this requirement [93,94,95].

### 4.4. Biochemical Analysis

Plasma melatonin concentration was determined using a commercially available enzyme immunoassay kit «ELISA Kit for Melatonin» (CEA908Ge, Cloud-Clone Corp. (Katy, TX, USA)) according to the manufacturer’s instructions, using an ELISA 200 analyzer (Yantai ADC, Beijing, China).

Biochemical parameters, including glucose, aspartate aminotransferase (AST), alanine aminotransferase (ALT), total protein, albumin, cholesterol, triglycerides, lactate dehydrogenase (LDH), and alkaline phosphatase (ALP), were measured using an automated biochemical analyzer (Cormay Accent 200, Cormay, Warszawa, Poland) with standard reagent kits (Cormay, Warszawa, Poland).

### 4.5. Histological and Morphometric Analysis

Liver fragments were fixed in 10% neutral buffered formalin, dehydrated in ascending concentrations of ethanol, and embedded in paraffin. Sections of 5 μm thickness were prepared and stained with hematoxylin and eosin (H&E) for general morphology assessment, and with Masson’s trichrome for connective tissue evaluation.

Morphometric analysis was performed using a Leica DM2500 microscope (Leica Microsystems, Wetzlar, Germany) equipped with a Leica DFC7000 T digital camera and Leica Application Suite X (LAS X) v. 4.13 image analysis software. The following parameters were measured in at least 50 fields of view per animal:

- Cross-sectional area of hepatocyte nuclei (S nucleus, μm^2^)
- Cross-sectional area of hepatocytes (S hepatocyte, μm^2^)
- Nuclear-cytoplasmic ratio (N/C ratio) was calculated as nuclear area divided by cytoplasmic area (cytoplasmic area = hepatocyte area—nuclear area)
- Proportion of binuclear hepatocytes (%) was determined by counting at least 1000 hepatocytes per animal
- Total liver preparations were used to obtain imprints, which were then stained with fuchsin-sulfurous acid according to the Feulgen method. Hepatocyte ploidy was calculated in ploidy units relative to the optical density of the stained diploid nuclei of small lymphocytes [96,97]
- Proportion of connective tissue (%) was measured in Masson’s trichrome-stained sections as the area of collagen fibers relative to the total tissue area [98]
- Proportion of hepatocytes with fatty degeneration (%) was assessed in H&E-stained sections
- Severity of steatosis was evaluated semi-quantitatively using a 4-point scale: 0—no steatosis, 1—mild (<30% of hepatocytes), 2—moderate (30–60%), 3—severe (>60%) [99,100].

### 4.6. Immunohistochemical Analysis

Immunohistochemical staining was performed on paraffin sections using the streptavidin-biotin peroxidase method. Sections were deparaffinized, rehydrated, and subjected to heat-induced antigen retrieval in citrate buffer (pH 6.0). Endogenous peroxidase activity was blocked with 3% hydrogen peroxide. Sections were incubated overnight at 4 °C with primary antibodies against the following markers:

- p16 (rabbit polyclonal, Abcam, Cambridge, UK, ab108349, dilution 1:200)
- p21 (rabbit polyclonal, Abcam, ab109520, dilution 1:150)
- p53 (mouse monoclonal, Abcam, ab1101, dilution 1:100)
- BMAL1 (rabbit polyclonal, Novus Biologicals, Centennial, CO, USA, NB100-2288, dilution 1:250)
- CLOCK (rabbit polyclonal, Abcam, ab3517, dilution 1:200)
- PER2 (rabbit polyclonal, Abcam, ab180655, dilution 1:150)
- Ki-67 (rabbit monoclonal, Abcam, ab16667, dilution 1:200)

After washing, sections were incubated with appropriate biotinylated secondary antibodies, followed by streptavidin-horseradish peroxidase complex. Visualization was performed using 3,3′-diaminobenzidine (DAB) as chromogen. Sections were counterstained with hematoxylin. For negative controls, primary antibodies were omitted. The expression index was calculated as the percentage of cells with positive staining relative to the total number of counted cells (at least 1000 cells per section) [101,102]. Sections in which primary antibodies were replaced with phosphate-buffered saline served as controls.

### 4.7. Electron Microscopy and Morphometric Analysis of Organelles

Liver samples (8 mm^3^) were fixed with 2.5% glutaraldehyde solution in phosphate buffer (pH 7.4), then additionally fixed in 1% osmium oxide solution (OsO4), dehydrated in ethanol, contrasted with 1% uranyl acetate solution in 70% ethanol during dehydration and embedded in an epon-araldite mixture according to the standard technique. Ultrathin sections obtained on a UC Enuity ultramicrotome (Leica Microsystems CMS GmbH, Wetzlar, Germany) were additionally contrasted with the use of Reynold’s lead citrate stain and viewed in a field emission scanning electron microscope HIMERA EM50X (CIQUTEK, Anhui, China), and photofixed. For analysis, uniform areas were selected: the intermediate zone of the liver lobules. The analysis was performed in 10 random fields of view (×10,000, area 25 μm^2^ each) to determine the numerical density of mitochondria and the sizes of hepatocytes and their nuclei, and in 10 random fields of view (×20,000, area 6.25 μm^2^ each) for micromorphometry of mitochondria and the Golgi complex [103,104].

The dissector method was used for stereometric studies [104]. All measurements were performed on the cross-section of mitochondria. The following parameters were assessed:

- Numerical density (units/μm^2^)—number of mitochondria per unit cytoplasmic area
- Cross-sectional area (μm^2^)
- Perimeter (μm)
- Number of cristae per mitochondrion
- Area-to-perimeter ratio
- Circularity index (calculated as 4π × area/perimeter^2^)

Morphometric analysis of the Golgi complex was performed on electron micrographs at a final magnification of ×20,000. The following parameters were assessed in at least 30 dictyosomes per animal:

- Number of dictyosomes per field of view
- Number of cisternae per dictyosome
- Profile area of the Golgi complex (μm^2^)

### 4.8. Statistical Analysis

Statistical analysis was performed using GraphPad Prism 8.0 software (GraphPad Software, Boston, MA, USA). Data are presented as mean ± standard deviation (SD). Normality of data distribution was assessed using the Shapiro–Wilk test. Comparisons between groups were performed using one-way analysis of variance (ANOVA) followed by Tukey’s post hoc test for multiple comparisons. For non-normally distributed data, the Kruskal–Wallis test with Dunn’s post hoc test was applied. Differences were considered statistically significant at *p* ≤ 0.05. Significance levels are indicated in the tables as follows: * *p* ≤ 0.05; ** *p* ≤ 0.001; *** *p* ≤ 0.0001 compared to the control group; ° *p* ≤ 0.05; °° *p* ≤ 0.001; °°° *p* ≤ 0.0001 compared to the DD group.

## 5. Limitations

Several limitations of this study should be acknowledged.

1. Species and age. The study was conducted on young adult male rats. Sex differences in circadian regulation and melatonin responses are well documented [10], and our findings may not fully extrapolate to females or aged animals.
2. Single time point. Animals were examined only at the end of the three-month experiment. A longitudinal design with intermediate time points would provide insights into the temporal sequence of pathological events and the dynamics of melatonin’s protective effects.
3. Melatonin administration regimen. Melatonin was given in drinking water (12 mg/L), corresponding to approximately 1–2 mg/kg/day. While effective, this route may not perfectly mimic the endogenous nocturnal surge. Additionally, the dose is supraphysiological, and dose–response studies would be valuable.
4. Mechanistic depth. Although we assessed key circadian and senescence markers, we did not perform functional assays (e.g., mitochondrial respiration, autophagy flux) or genetic manipulations (e.g., clock gene silencing) to establish causality. The observed associations, though robust, remain correlational.
5. We did not include a recovery group, in which animals would be returned to normal lighting after DD, to assess reversibility without melatonin treatment. Such a group would clarify whether melatonin accelerates recovery or is required for reversal.
6. Despite these limitations, the study provides a comprehensive, multi-level characterization of desynchronosis-induced liver pathology and demonstrates the powerful protective effect of melatonin. Future studies should address the identified gaps, including the detailed analysis of hepatic drug-metabolizing enzymes and cytochrome P450 (CYP) isoforms, and explore translational applications in human populations. These findings establish a strong foundation that rigorously defines the parameters of the phenotype; future studies should address the identified gaps to build upon this groundwork and explore translational applications.

## Figures and Tables

**Table 1:** Biochemical parameters of rat blood plasma after 3 months of the experiment (age 6 months). Data are presented as mean ± SD. DD: Dark deprivation group; DD + Mel: Dark deprivation + Melatonin group.

Parameter	Control (n = 30)	DD (n = 30)	DD + Mel (n =30)
**Melatonin, pg/mL**	77.29 ± 8.59	15.18 ± 3.27 ***	65.58 ± 11.90 °°°
**Glucose, mmol/L**	6.39 ± 0.49	4.64 ± 0.89 ***	5.99 ± 0.87 °°°
**AST, U/L**	79.64 ± 9.68	90.38 ± 11.67 *	74.40 ± 5.26 °°
**ALT, U/L**	42.24 ± 5.39	50.23 ± 6.28 ***	42.72 ± 4.38 °°°
**Total protein, g/L**	64.09 ± 5.14	47.27 ± 4.57 ***	63.56 ± 7.28 °°°
**Albumin, g/L**	49.03 ± 5.30	33.24 ± 4.06 ***	48.04 ± 3.61 °°°
**Cholesterol, mg/dL**	58.59 ± 4.83	91.54 ± 10.70 ***	60.6 ± 5.09 °°°
**Triglycerides, mmol/L**	0.86 ± 0.04	1.50 ± 0.14 ***	0.86 ± 0.12 °°°
**LDH, U/L**	683.1 ± 53.72	947.7 ± 93.17 ***	750.0 ± 53.16 *** °°°
**ALP, U/L**	99.77 ± 10.79	159.0 ± 8.28 ***	95.99 ± 13.76 °°°

Note. * *p* ≤ 0.05; *** *p* ≤ 0.0001—compared to the control group parameters. °° *p* ≤ 0.001; °°° *p* ≤ 0.0001—compared to the DD group parameters.

**Table 2:** Morphometric parameters of hepatocytes and liver structure of rats after 3 months of the experiment. Data are presented as mean ± SD. DD: Dark deprivation group; DD + Mel: Dark deprivation + Melatonin group.

Parameter	Control (n = 30)	DD (n = 30)	DD + Mel (n = 30)
**S nucleus, µm^2^**	41.72 ± 8.24	43.07 ± 5.34	40.31 ± 10.08
**S hepatocyte, µm^2^**	186.50 ± 28.51	265.82 ± 43.01 ***	180.60 ± 25.83 °°°
**N/C ratio**	0.23 ± 0.05	0.16 ± 0.021 ***	0.23 ± 0.06 °°°
**Proportion of binuclear hepatocytes, %**	10.08 ± 1.63	5.82 ± 0.68 ***	12.21 ± 1.47 *** °°°
**Mean ploidy of hepatocytes, n**	2.90 ± 1.4	3.22 ± 1.70	3.20 ± 1.84
**Proportion of connective tissue, %**	2.99 ± 0.27	6.03 ± 0.70 ***	3.04 ± 0.32 °°°
**Proportion of hepatocytes with fatty degeneration**	3.47 ± 0.7	9.13 ± 0.67 ***	4.09 ± 0.27 *** °°°
**Severity of steatosis**	0.40 ± 0.30	2.80 ± 0.70 ***	0.90 ± 0.50 °°°

Note. *** *p* ≤ 0.0001—compared to the control group parameters. °°° *p* ≤ 0.0001—compared to the DD group parameters.

**Table 3:** Expression of molecular markers in rat hepatocytes after 3 months of the experiment. Data are presented as mean ± SD. DD: Dark deprivation group; DD + Mel: Dark deprivation + Melatonin group.

Marker	Control (n = 30)	DD (n = 30)	DD + Mel (n = 30)
**p16**	0.51 ± 0.11	12.34 ± 2.35 ***	1.07 ± 0.28 °°°
**p21**	1.12 ± 0.14	7.58 ± 1.41 ***	1.87 ± 0.38 °°°
**p53**	2.19 ± 0.46	3.97 ± 0.65 **	2.24 ± 0.44 °°
**BMAL1**	60.68 ± 5.85	16.55 ± 5.34 ***	60.32 ± 18.44 °°°
**CLOCK**	54.61 ± 5.27	15.72 ± 5.27 ***	68.0 ± 18.48 °°°
**PER2**	31.09 ± 6.32	40.06 ± 10.53 ***	30.06 ± 8.75 °°
**Ki-67**	1.81 ± 0.05	3.50 ± 0.03 ***	1.93 ± 0.17 °°°

Note. ** *p* ≤ 0.001; *** *p* ≤ 0.0001—compared to the control group parameters. °° *p* ≤ 0.001; °°° *p* ≤ 0.0001—compared to the DD group parameters.

**Table 4:** Morphometric parameters of rat hepatocyte mitochondria after 3 months of the experiment. Data are presented as mean ± SD. DD: Dark deprivation group; DD + Mel: Dark deprivation + Melatonin group.

Parameter	Control (n = 30)	DD (n = 30)	DD + Mel (n = 30)
**Numerical density, units/µm^2^**	1.69 ± 0.26	1.44 ± 0.23	1.71 ± 0.20 °
**Cross-sectional area, µm^2^**	0.319 ± 0.04	0.314 ± 0.088 **	0.321 ± 0.041
**Perimeter, µm**	1.93 ± 0.16	1.98 ± 0.13	1.94 ± 0.15
**Number of cristae, pcs**	23.45 ± 3.58	21.52 ± 3.88 **	24.01 ± 3.59
**Area-to-perimeter ratio**	0.165 ± 0.031	0.158 ± 0.024	0.165 ± 0.022
**Circularity index**	0.93 ± 0.04	0.97 ± 0.06	0.94 ± 0.05

Note. ** *p* ≤ 0.001—compared to the control group parameters. ° *p* ≤ 0.05—compared to the DD group parameters.

**Table 5:** Morphometric parameters of the Golgi complex in rat hepatocytes after 3 months of the experiment. Data are presented as mean ± SD. DD: Dark deprivation group; DD + Mel: Dark deprivation + Melatonin group.

Parameter	Control (n = 30)	DD (n = 30)	DD + Mel (n = 30)
** Number of dictyosomes per field of view, units **	4.02 ± 0.30	3.91 ± 0.38	3.98 ± 0.31
** Number of Golgi complex cisternae, pcs **	8.07 ± 0.99 *	7.08 ± 1.03 *	8.11 ± 1.17 ***
** Profile area of the Golgi complex, µm^2^**	3.27 ± 0.48 °°	2.75 ± 0.38 °°	3.33 ± 0.28 °°

Note. * *p* ≤ 0.05; *** *p* ≤ 0.0001—compared to the control group parameters. °° *p* ≤ 0.001.

## Data Availability

The original contributions presented in this study are included in the article. Further inquiries can be directed to the corresponding author.
